# Design and *in silico* validation of donor DNA for RNA-guided recombinase-mediated knockout of *mstnb* gene in *Labeo rohita*

**DOI:** 10.1371/journal.pone.0352166

**Published:** 2026-07-15

**Authors:** Lukram Sushil Singh, Porkodi Murugesan, Gowhar Iqbal, Nagavara C. Nidarshan, Harini Gunasekaran, Ad. Viralkumar, Kiran D. Rasal, Manoj P. Brahmane, Sunil K. Nayak, Arvind A. Sonwane, Mukunda Goswami

**Affiliations:** Division of Fish Genetics and Biotechnology, ICAR-Central Institute of Fisheries Education, Mumbai, Maharashtra, India; Bangladesh Agricultural University, BANGLADESH

## Abstract

Myostatin b (*mstnb*), a key negative regulator of skeletal muscle growth, represents a promising target gene for genome editing aimed at enhancing growth performance and aquaculture productivity. The present study aimed to design, construct and *in silico* validate a donor DNA carrying a single-nucleotide substitution that introduces a premature stop codon in the *mstnb* gene of *Labeo rohita* for RNA-guided recombinase (RGR) platform-mediated genome editing. For this purpose the *mstnb* gene sequence was retrieved from the NCBI database and analysed to identify an appropriate target site within exon 1. A targeted single nucleotide substitution from guanine to thymine (G > T) was strategically planned into the donor DNA upstream of the native stop codon to convert the glycine codon (GGA) into a premature stop codon (TGA). This was achieved by identifying RGR target sites flanking the intended mutation site and designing specific primers to amplify and clone the DNA fragment. The target 600 bp DNA fragment encompassing the mutation site flanked by two RGR target sites was successfully amplified and ligated into the pJET1.2 cloning vector and confirmed through Sanger sequencing. Site-directed mutagenesis successfully introduced the intended nucleotide substitution, which was subsequently confirmed by Sanger sequencing. Computational analyses using InterPro, ColabFold, SWISS-MODEL and CYS_REC predicted that the introduced nonsense mutation would generate a truncated *Mstnb* protein, resulting in the loss of conserved TGF-β domains, reduced structural stability, and impaired cytokine activity. Structural modelling further revealed disruption of the C-terminal β-sheet structure, reduced stereochemical quality, altered QMEAN Z-scores, and loss of cysteine residues, collectively indicating impaired protein folding and reduced structural stability. These findings suggest that the engineered mutation is likely to abolish the functional activity of the *mstnb* gene, thereby providing a validated donor DNA construct for precise RGR-mediated genome editing in *L. rohita*. Future studies will focus on the experimental validation of the engineered donor DNA construct through RGR-mediated genome editing, followed by functional characterization in *L. rohita*.

## Introduction

Growth is a fundamental biological process primarily governed by skeletal muscle development through a multistep process known as myogenesis, which is regulated by various negative regulatory factors. Myogenesis is controlled by myogenic regulatory factors, including myogenin, MyoD, Myf5, and MRF4/6, along with paired box family proteins (Pax3 and Pax7) and myocyte enhancer factor 2 (MEF2) family proteins, which collectively regulate muscle-specific gene expression and coordinate skeletal muscle development [1,2].Moreover, myostatin (*mstn*), or growth and differentiation factor 8 (GDF8), belongs to the transforming growth factor-β (TGF-β) superfamily, functions as a negative regulator of skeletal muscle growth, limiting the number and size of skeletal muscle fibers. Several researches were carried out for myostatin and to study its role in muscle growth in livestock [2].

Myostatin is highly conserved across several vertebrate species, and the first genomic organization for the murine species was provided by McPherron *et al*. [3]. It was identified for the first time that muscular hypertrophy known as double-muscle phenotype in Belgian Blue and Piedmontese cattle was due to functional loss of myostatin through natural mutation [4]. Later, a similar loss-of-function mutation was observed in dogs, sheep, rabbits, and even in rare cases in human highlighting the evolutionary role in regulating skeletal muscle growth. In teleosts, the two paralogs of the myostatin gene, myostatin-b (*mstnb*) and myostatin-a (*mstna*), are involved in muscle development and immune function, respectively [5]. During embryogenesis, myostatin is expressed exclusively in skeletal muscle, but during the adult stage, lower levels of expression are visible in other tissues, including heart, adipose tissue, and mammary gland, indicating its involvement in large physiological processes [6,7]. Improvement of livestock output requires understanding of the molecular genetics of myostatin and its tissue-specific bioactivity [8].

Gene targeting began in the 1970s with homologous recombination but was limited by low efficiency and complexity. This led to the development of nuclease-based tools such as meganucleases, ZFNs, TALENs, and later CRISPR-Cas9, which enabled efficient and targeted DNA cleavage but depend on cellular repair mechanisms, often causing off-target effects [9–12]. However, recombinase-mediated genome editing offers a safer and more precise alternative. It functions independently of endogenous DNA repair pathways reducing genotoxic risks [13]. The site-specific recombinases (SSRs) specifically catalyse cleavage, strand exchange and re-ligation of two double-stranded DNA sequences, resulting a single predetermined outcome [14]. However, they have inherent, sequence specificity, and limitation in higher eukaryotic genomes with limited target sites or need pre-introduced sites. Hence, recombinases have been evolved experimentally and made ‘hyperactivated’ to create broader genomic target sites instead of a single site [15,16]. These recombinases have been fused with zinc finger and TALE DNA-binding proteins to create programmable zinc finger and TALE recombinases, expanding the range of targetable recognition sequences, thereby increased the applicability of recombinase action-based GE technology [17,18]. HDR-based strategies enable the introduction of precise and predefined genetic modifications using donor DNA templates. However, HDR efficiency is often limited due to low recombination frequency and challenges in donor design [19].

Recently, RNA-guided recombinase (RGR), a hyperactivated recombinase-mediated genome editing platform where the nuclease-null or dead Cas9 (dCas9) fused with the hyperactivated recombinases to enable simple and programmable genome editing has been described. RGR platforms are based on unique recognition sites that range in length from 76 to 80 base pairs. These sites include two guide RNA binding sequences (20-bp) and a 20-bp degenerate recombinase pseudo-core site that is surrounded by 5–7-bp spacers. The entire whole central domain is flanked by 3-bp Protospacer Adjacent Motifs (PAMs) at both ends, ensuring precise recombinase activity and gRNA-directed targeting. These platforms utilize RNA-guided processes to mediate recombination, allowing precise genome alterations independent of endogenous cellular machinery and producing a single well-defined product with high specificity and minimal genomic alterations [20–22]. The platform allows addition or removal of homologous targeted nucleotides through RGR-mediated cassette exchange (RGR-MCE). This is characterized by a donor DNA with a pair of RGR target sites flanking the targeted locus, directing recombination between genomic DNA and engineered donor DNA, enabling insertion of an upstream stop codon in the genomic DNA to achieve efficient gene knockout and transcriptional disruption [23].

Aquaculture has seen significant growth in production and economic value, driven by rising demand for fish products. To enhance the growth rates of aquatic species, several genetic improvement strategies were used to target the growth genes. The myostatin gene is a potential target gene where extensive research has been focused on. It is known to inhibit muscle growth, making it a prime candidate for genetic manipulation to improve growth performance in aquaculture species. The disruption of the myostatin gene can lead to increased muscle growth in fish, enhancing overall growth [24,25]. Aquaculture represents an expanding sector within food industry that supports food and nutritional security worldwide. Freshwater aquaculture, dominated by carps with rohu (*Labeo rohita*) alone contribute 15% to the world’s freshwater aquaculture production [26]. Study on genetic manipulation on rohu’s *mstnb* gene will offer valuable insights into the gene for enhancing muscle growth in rohu. This study aims to construct donor DNA that carries a premature stop codon for the *mstnb* gene knockout using RGR platform in *L. rohita* along with computational analysis of predicted protein following truncation due to premature stop codon insertion.

## Materials and Methods

### Ethics statement

The experiments conducted in the present study were carried out in accordance with the ethical guidelines for the use of fish prescribed by the Committee for Control and Supervision of Experiments on Animals (CCSEA), a statutory committee under the Department of Animal Husbandry and Dairying (DAHD), Ministry of Fisheries, Animal Husbandry and Dairying (MoFAH&D), Government of India (vide 524/GO/ReRcBiBit/S/02/CCSEA). The experimental protocol was also reviewed and approved by the Board of Studies (BoS) of the Division of Fish Genetics and Biotechnology, ICAR–Central Institute of Fisheries Education (ICAR-CIFE), Mumbai, India (vide FBT-PB2–06).

### Localization of single nucleotide substitution in the *mstnb* gene sequence

The experiment was conducted at Fish Genetics and Biotechnology Laboratory, ICAR-Central Institute of Fisheries Education (CIFE), Mumbai, India. The nucleotide sequence of the rohu myostatin b (*mstnb*) gene was acquired from the NCBI database, having Reference Sequence: NC_066877.1 (REGION: 27438698..27442252). The *mstnb* nucleotide sequence was analyzed using DNASTAR EditSeq software to identify the longest open reading frame (ORF). Codons were manually examined to identify a suitable single nucleotide substitution capable of introducing a premature stop codon upstream of the native stop codon in the coding region. Based on this analysis, a nucleotide substitution site at position 404 bp within the first exon (E1) of the *mstnb* gene sequence was selected. Then the recognition sequence 5′-CCN_(74)_GG-3′ of the hyperactivated Sin recombinase-based RGR platform were used as input into the DNA Pattern Find tool (http://bioinformatics.org/sms2/dna_pattern.html) to identify the RGR target sites in the rohu *mstnb* gene. A pair of RGR target sites were selected that flanked the targeted mutation site. Then a pair of primer was designed (amplicon size 600 bp) flanking both the RGR target site and targeted mutation site. The quality of designed primers (Table 1) was verified by using IDT’s OligoAnalyzer tool.

**Table 1:** Primers used for PCR amplification and site-directed mutagenesis (SDM).

Primers for PCR amplification	oAS251	CCATAGCAAATCAAATCAAACATCGC
	oAS252	AATCAGTAAACGAGAACTATCTTCGC
Primers for Q5 SDM reaction	oAS253	CAGCAAGGATtGAGCTCTGGAAG
	oAS254	TCATCCCCCAGAACATCG

### DNA isolation and PCR amplification

The *L. rohita* fish brought from the ICAR- Central Institute of Fisheries Education, Powarkheda, Narmadapuram, Madhya Pradesh, India was euthanized using clove oil in accordance with standard ethical guidelines for animal handling and care. Muscle tissues were carefully taken under sterile conditions and DNA extraction was performed in triplicate biological samples. Among the isolated DNA samples, the sample showing the best quality and integrity was selected for subsequent downstream process. DNA was isolated following the protocol with minor modification described by Sambrook and Russell [27]. Samples were homogenised and incubated overnight at 55°C in lysis buffer using a water bath. After lysis, DNA was extracted using phenol-chloroform and subsequently precipitated, washed, and suspended in TE buffer. The concentration and purity of DNA were evaluated using NanoDrop spectrophotometer (Thermo Scientific). The DNA yield ranged from approximately 600.7 to 750.8 ng/μL across the samples and the results were recorded using a gel documentation system (OmegaLum G, Aplegen).

The targeted region of the *mstnb* gene was amplified using Thermo Scientific Phire Hot Start II DNA Polymerase along with specific primers listed in Table 1. Briefly, PCR amplification was carried out in a total reaction volume of 12.5 µl containing 2.5 µl of 5 × Phire Reaction Buffer, 0.25 µl of dNTP mix, 0.5 µl each of forward (oAS251) and reverse (oAS252) primers, 2.5 µl of template DNA (100 ng/µl), 0.25 µl of Phire DNA Polymerase, and 6.0 µl of nuclease-free water. The amplification was performed following conditions of an initial denaturation – 98^°^C for 30 seconds, denaturation – 98^°^C for 5 seconds, annealing- 59^°^C for 5 seconds, extension – 72^°^C for 10 seconds, with final extension – 72^°^C for 1 minute before the reaction was held at 4^°^C. The amplified product was confirmed in 1% agarose gel electrophoresis followed by elution using the QIAquick Gel Extraction Kit (Qiagen, Cat. No. 28704). The eluted DNA was reconfirmed in 1% agarose gel electrophoresis.

### Cloning and transformation

Cloning was performed by ligating the eluted PCR product into the cloning vector (pJET1.2 blunt-end vector; Thermo Scientific, Cat. No. K1231) using T4 DNA ligase according to the manufacturer’s protocol. Ligation was carried out in a total reaction volume of 10 µl containing 5.0 µl of 2 × Reaction Buffer, 1.5 µl of purified PCR product, 0.5 µl of pJET1.2 cloning vector, 0.5 µl of T4 DNA ligase, and 2.5 µl of nuclease-free water. The ligation mixture was incubated at 22°C for 5 min. The ligated product was transformed into *DH5α E. coli* competent cells and cultured on ampicillin-containing Luria-Bertani (LB) agar plates. Recombinant clones were confirmed through colony PCR using DreamTaq DNA polymerase (Thermo Scientific, Cat. No. EP0701) following the manufacturer’s instructions. Colony PCR was performed in a total reaction volume of 7.5 µl containing 1.25 µl of 10 × DreamTaq buffer, 0.25 µl of dNTP mix (10 mM), 0.25 µl each of forward (oAS251) and reverse (oAS252) primers, 0.2 µl of DreamTaq DNA polymerase, and 5.3 µl of DEPC-treated water. The amplification was carried out for 30 reaction cycle with an initial denaturation at 95°C for 3 min, followed by denaturation at 95°C for 30 s, annealing at 59°C for 30 s, and extension at 72°C for 30 s. A final extension was performed at 72°C for 5 min, and the reaction was subsequently held at 4°C. Confirmed positive colonies were inoculated into 5 ml ampicillin LB broth medium and incubated overnight at 180 rpm and 37°C. Subsequently, plasmids were isolated using the QIAprep Spin Miniprep Kit (Qiagen, Cat. No. 27104). The plasmid integrity was checked through agarose gel electrophoresis, and the presence of the insert was subsequently confirmed by Sanger sequencing.

### Site-directed mutagenesis (SDM)

The confirmed plasmid DNA containing insert went site-specific mutagenesis through Q5 Site-Directed Mutagenesis Kit (NEB, Cat. No.: E0554S), using Q5 Hot Start High-Fidelity DNA Polymerase and a primer pair with a custom mutagenic primers (Table 1). The mutagenic primer was engineered to incorporate a single base-pair mismatch at the desired site with at least 10 nucleotides complementary to the insert at the 3′ terminus of the primer, to facilitate substitution in the template the insert donor DNA. Whereas the other primer was oriented in the opposite direction, complementary to the plasmid, to undergo exponential amplification. The exponential amplification of the target was carried out using reaction mixture as shown in Table 2, under the following conditions: denaturation at 98°C for 10 seconds; annealing at 64°C for 30 seconds; and extension at 72°C for 15 seconds, for 25 cycles. The amplified product was treated at room temperature with Kinase-Ligase-DpnI (KLD) enzyme mix to digest the parental template DNA and enable efficient rapid circularization of the mutated plasmid. The resulting construct was transformed into chemically-competent *E. coli* by following the manufacturer’s protocol and then cultured in an ampicillin LB agar plate overnight at 37°C. A healthy colony was taken and cultured overnight in 5 ml LB broth with ampicillin in a shaker incubator at 200 rpm at 37°C. Plasmids were isolated followed by Sanger sequencing to confirm the successful incorporation of the substitution mutation.

**Table 2:** Composition of the PCR reaction mixture used for SDM amplification with Q5 Hot Start High-Fidelity 2×  Master Mix using primers oAS253 and oAS254.

Components	Volume (µl)
Q5 Hot Start High Fidelity 2X Master Mix	6.25 µl
Forward Primer (oAS253)	0.625 µl
Reverse Primer (oAS254)	0.625 µl
Template (15 ng/ul)	0.5 µl
Nuclease-free water	4.5 µl
Total	12.5 µl

### Domain prediction and homology modelling of mstnb upon truncation

Computational evaluation of the predicted peptide sequences of mutated *mstnb* and wild-type *mstnb* sequences of rohu was done by using (InterPro database) (https://www.ebi.ac.uk/interpro/) to compare the predicted domains and important structural differences between them. InterPro integrates 13 different protein signatures (predictive models) from multiple databases [28]. The InterPro consortium, which integrates several databases, was employed to categorize proteins into families and domains and to infer functional properties based on these classifications [29].

The 3D structural analysis of the wild-type *mstnb* and mutated *mstnb* sequences were conducted using ColabFold-AF2 platform to identify the structural differences after mutation [30]. Further *in silico* comparative analysis of predicted protein quality was performed using SWISS-MODEL [31]. The cysteine residues and potential disulfide bonds in the predicted protein structures were identified using the CYS_REC program implemented in the SoftBerry server (http://www.softberry.com/cgi-bin/programs/propt/cys_rec.pl). The presence of cysteine residues that involved in disulphide bonds formation ultimately explain the structural stabilization of the predicted protein structure [32].

## Results

The myostatin b (*mstnb*) gene is located on chromosome 9 of *L. rohita.* The gene spans 3.55 kb and comprises three exons. It is located between positions 27,438,698 bp and 27,442,252 bp on chromosome 9 of rohu (*Labeo rohita*), within the 42,740,749 bp whole-genome shotgun sequence (Accession No. NC_066877.1). The target region for nucleotide substitution was identified at 404 bp position in the first exon of the *mstnb* sequence by using the DNA star EditSeq software. It was planned here that, after the substitution, the first guanine in the GGA codon will be replaced by thymine to create the TGA codon, an upstream stop codon. As a result, the glycine amino acid will be replaced by a stop codon, producing a nonsense mutation that will interfere *mstnb* gene expression.

**Fig 1:**
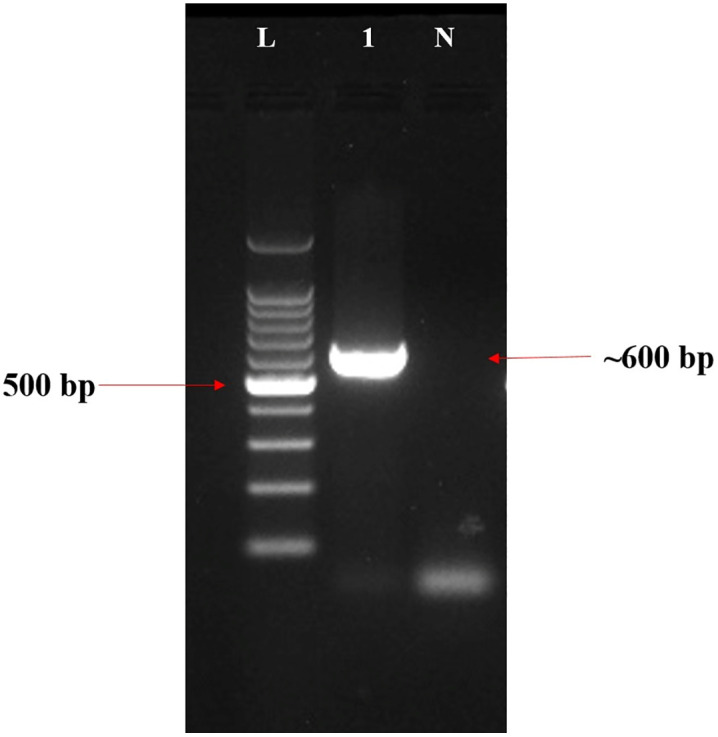
Agarose gel electrophoresis showing amplification of the rohu *mstnb* gene fragment using primers oAS251 and oAS252. Lane L: 100 bp Plus DNA ladder; Lane 1: amplified *mstnb* fragment; Lane N: non-template control.

**Fig 2:**
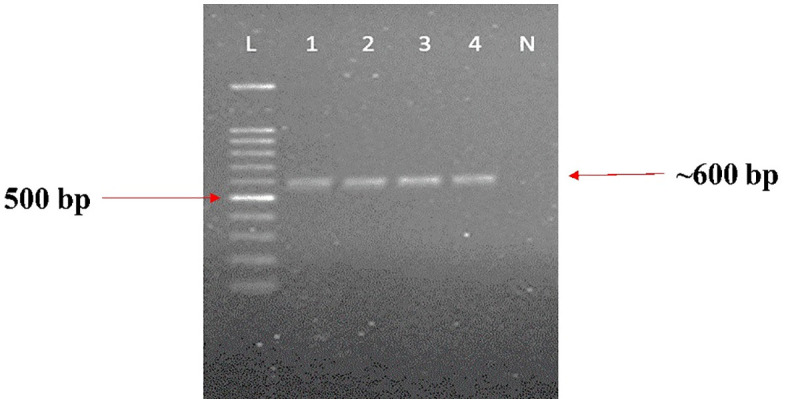
Agarose gel electrophoresis (1%) showing colony PCR results after transformation. L: 100 bp Plus DNA ladder; Lanes 1-4: individual colonies; Lane N: non-template control.

**Fig 3:**
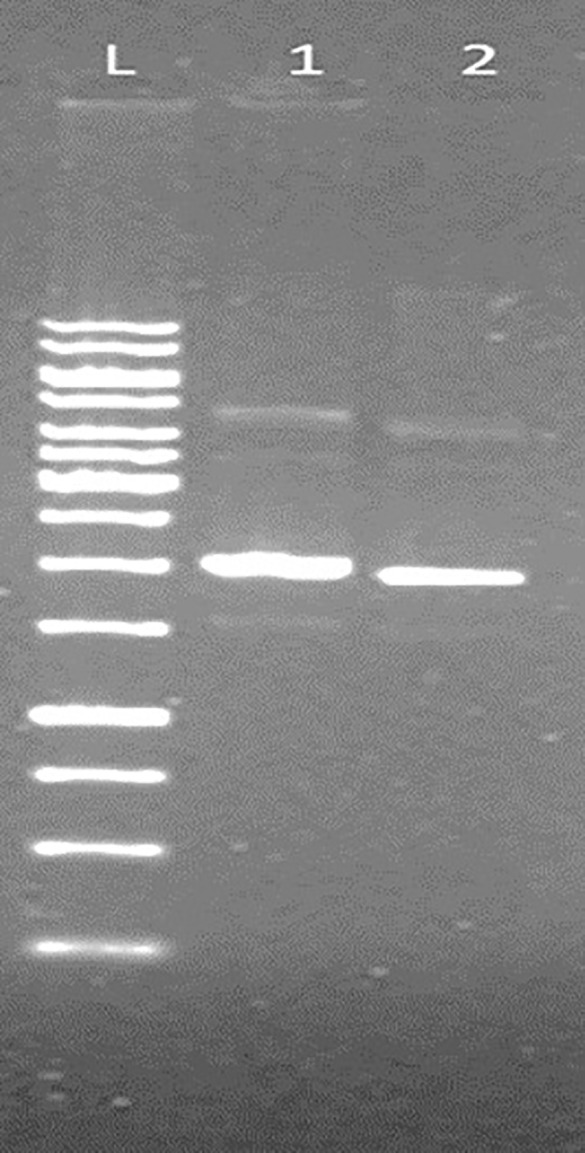
Plasmid isolation from confirmed colonies. Lane L: 1 kb DNA ladder; Lanes 1 and 2: Plasmid DNA.

**Fig 4:**
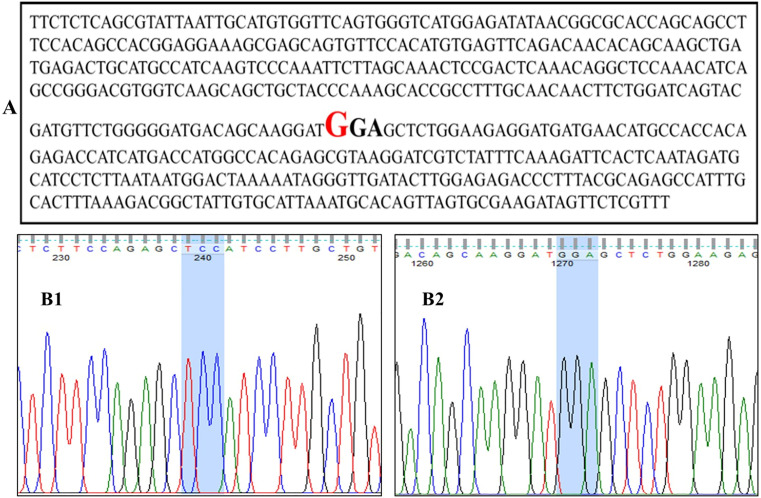
Sanger sequencing analysis confirming integration of the targeted sequence into the vector. (A) Sequence of the targeted region showing the location for point mutation with the nucleotide to be substituted (G) highlighted in red. (B1) Forward, and (B2) reverse sequence chromatograms confirming the location of targeted nucleotide substitution.

The DNA was amplified by using primer oAS251 and oAS252 and amplicon size of 600 bp was observed (Fig 1). Gel elution was performed and the eluted product was cloned in pJet1.2 vector and was used to transform competent *E. coli*. Colony PCR was performed to confirm the cloning of 600 bp amplicon (Fig 2). The confirmed colonies were inoculated in 5 ml LB broth cultures and plasmids were isolated (Fig 3). Insertion of the target sequence into the vector was further confirmed through Sanger sequencing (Figs 4A and B2). The sequencing results are provided as Supplementary Figure S1 (S1 Fig).

**Fig 5:**
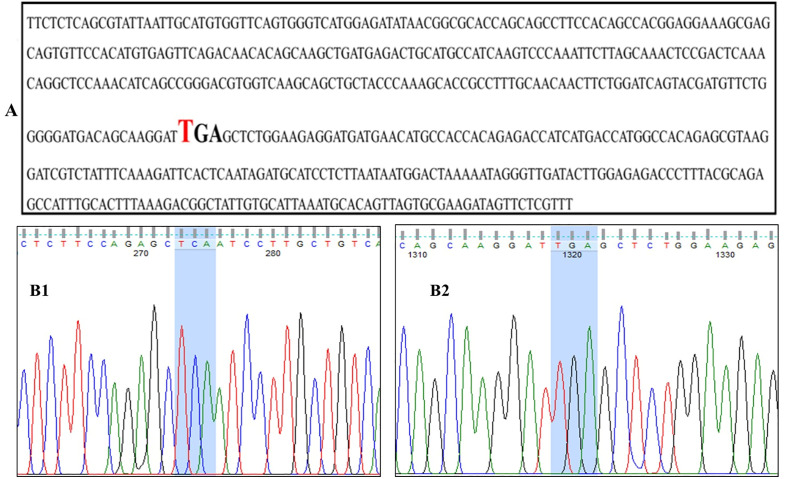
Sanger sequencing results following site-directed mutagenesis (SDM). (A) DNA sequence showing the targeted nucleotide substitution from G to T (highlighted in red), resulting in the formation of a premature stop codon (TGA) without additional sequence errors. (B1) Forward, and (B2) reverse sequence chromatograms confirming the successful incorporation of the targeted nucleotide substitution (G > T) after SDM.

Sequence verified plasmid was used to performed single nucleotide substitution through site-directed mutation (SDM). Many colonies were observed after SDM, and the plasmids were isolated from the broth-cultured colonies for sequencing. The sequencing after SDM showed precise introduction of single nucleotide mutation substituting guanine nucleotide by thymine (G > T) at the target site. The chromatograms showed proper mutation site with a clear peak of thymine nucleotide replacing the guanine nucleotide without any off-target mutation or alteration in the sequence, validating the precision of the experimental approach (Figs 5A and B2). Sequencing results are provided as Supplementary Figure S2 (S2 Fig).

**Fig 6:**
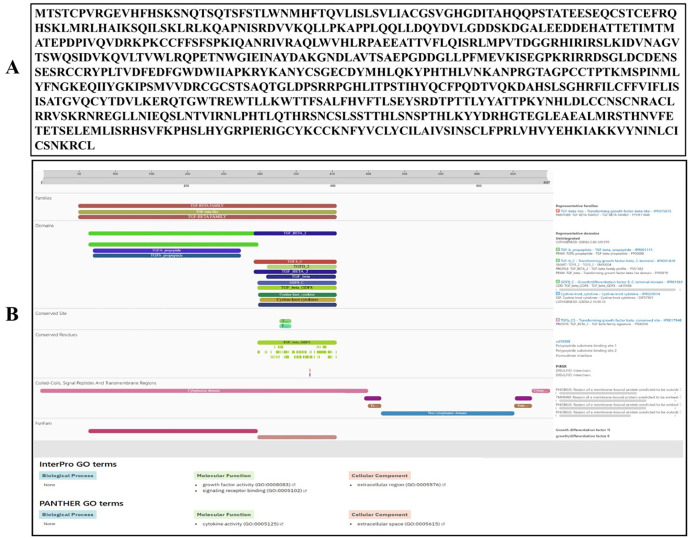
Protein domain structure prediction of *L. rohita* *Mstnb* protein. (A) Input predicted amino acid sequence of the wild-type *Mstnb* protein used for analysis. (B) Predicted protein domain structure of the wild-type *Mstnb* protein generated through *in silico* analysis.

**Fig 7:**
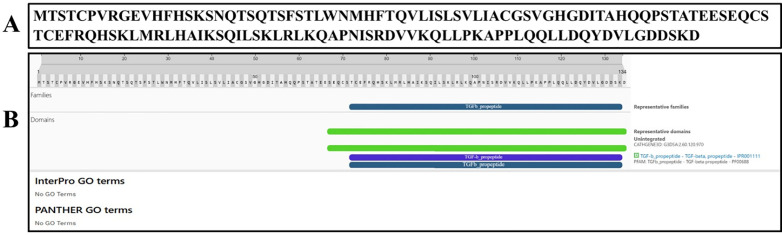
Protein domain structure prediction of *L. rohita.* (A) Input predicted amino acid sequence of truncated *Mstnb* protein; (B) Output protein domain structure prediction of wild-type *Mstnb* protein.

Interpro analysis of the wild-type *mstnb* protein was performed using the predicted amino acid sequence as input (Fig 6A). It revealed that the myostatin is associated with molecular functions of growth factor activity (GO:0008083) and signaling receptor binding (GO:0005102) in the extracellular region (GO:0005576). It is also involved in cytokine activity (GO:0005125) within the extracellular space (GO:0005615) (Fig 6B). Furthermore, the protein contains highly conserved TGF-β propeptide and TGF-β cytokine domains, highlighting the evolutionary conservation of its structural and functional features. These findings are in agreement with the well-established of *mstn* as a negative regulator of muscle growth, where the propeptide domain confers latency to the protein until the subsequent proteolytic cleavage releases the active C-terminal cytokine domain. However, upon mutation, the protein sequence was predicted to become truncated, and the truncated protein sequence was used as the input for further analysis (Fig 7A). This truncation predicted a significant disruption of cytokine activities, along with the loss of the TGF-β propeptide domain (Fig 7B). Consequently, the truncated protein may fail to undergo proper proteolytic cleavage and activation of the C-terminal cytokine domain.

**Fig 8:**
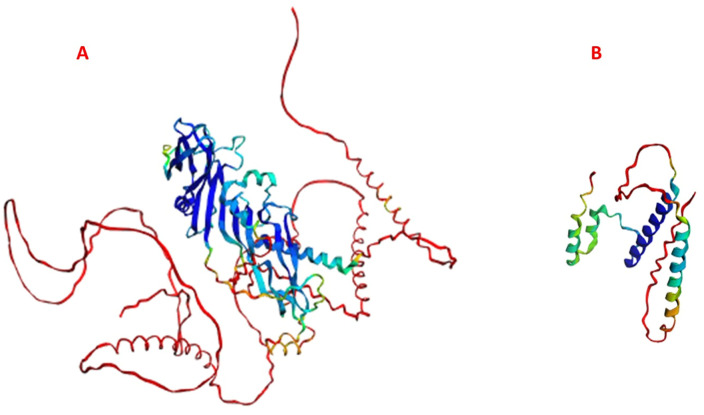
Predicted protein structure using ColabFold v1.6.1: AlphaFold2. (A) Wild-type Mstnb having C-terminal chain (β sheets); (B) truncated *Mstnb*, loss of C-terminal region.

**Fig 9:**
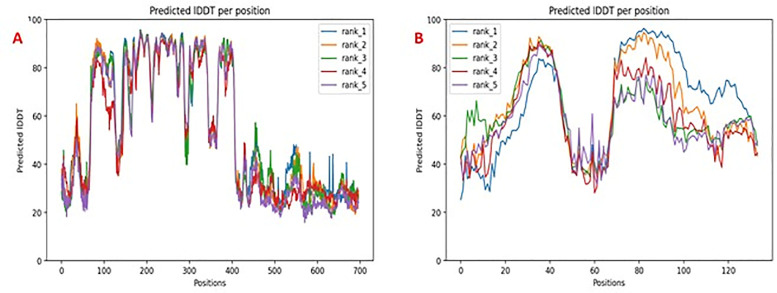
Comparative pLDDT analysis of predicted protein structure. (A) Wild-type Mstnb showing high-confidence structured core; (B) Truncated Mstnb showing disrupted folding.

**Fig 10:**
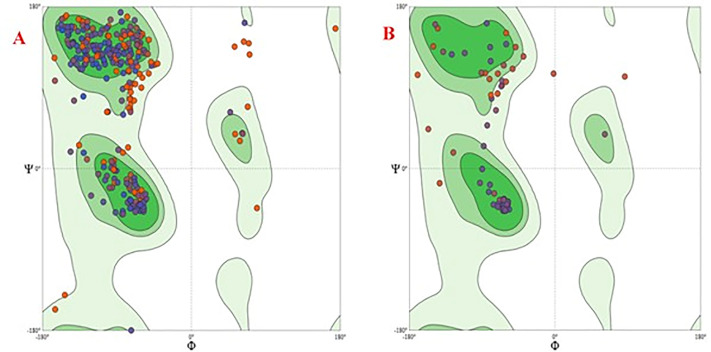
Ramachandran plot of *Mstnb* (A) wild-type *Mstnb* showing maximum residues in the favoured regions; and (B) mutated *Mstnb* with loss of residues from favoured regions.

**Fig 11:**
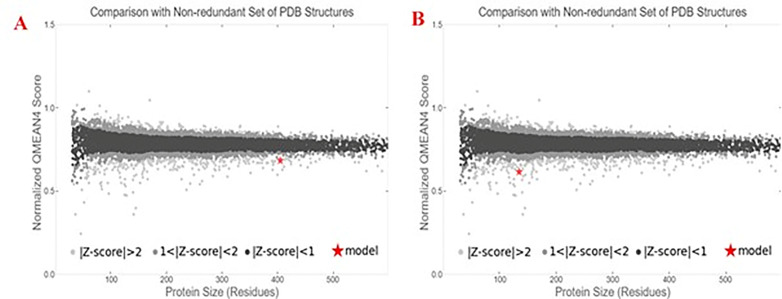
QMEAN score placed on a scatter plot against the score of the non-redundant set. The red star represents the modelled protein structure. (A) wild-type *Mstnb*; and (B) truncated *Mstnb*.

ColabFold v1.6.1 platform was used to generate the 3D structure of *mstnb* (wild-type and truncated as shown in Fig 8A and B). The truncation of *mstnb* led to a pronounced disruption of β-sheet structures associated with the C-terminal domain. Comparative pLDDT analysis demonstrated that the wild-type *mstnb* protein possesses a continuous and well-defined high-confidence structural core, while the truncated variant displays impaired folding, as evidenced by fragmented high-confidence regions and elevated intrinsic disorder (Fig 9A and B). The loss of structural integrity following truncation, suggests impaired protein folding and a consequent reduction in the functional capacity of the truncated protein. Further the quality of the predicted protein structure was evaluated using the SWISS-MODEL server. The wild-type *mstnb* structure exhibited a QMEANDisCo global score of 0.63 ± 0.05, indicating moderate model reliability, whereas the truncated variant showed a slightly higher score of 0.68 ± 0.08. Ramachandran plot analysis revealed that 89.28% of residues in the wild-type model were located within the favored regions, with only 1.61% classified as outliers. In contrast, the truncated model contained 83.33% of residues in the favored regions and 2.94% outliers, indicating increased backbone distortion and reduced stereochemical quality following truncation (Fig 10A and B; Table 3). MolProbity assessment further supported these observations, with scores of 1.38 and 1.67 for the wild-type and truncated models, respectively. The lower MolProbity score of the wild-type model indicates superior stereochemical geometry and overall structural quality compared with the truncated variant. The QMEAN Z score (Qualitative Model Energy Analysis) was used to assess the structural quality of the predicted protein model by comparing it against a non-redundant dataset of experimentally determined high-resolution structures available in the Protein Data Bank (PDB). In the wild-type, the *mstnb* model is positioned at approximately 400 residues and clusters within the central region of PDB structures (1 < |Z-score| < 2), which suggests a good-quality indicating a reliable structural model. In contrast, the truncated model falls within the 100–200 residue range and is shifted further downward (|Z-score| > 2) on the normalized QMEAN Z-score plot, indicating suggesting a greater deviation from experimentally resolved protein structures (Fig 11A and B). Collectively, these findings suggest that the truncation disrupts key secondary structural elements involved in dimerization, thereby compromising the intramolecular interface required for the formation of functional dimers.

**Table 3:** Result summary of the Ramachandran plot emphasizing the structural difference between wild-type and truncated *Mstnb.*

Description	Residues in favoured region	Residue in outlier region	MolProbity Score
Wild-type *Mstnb*	89.28%	1.61%	1.38
Truncated *Mstnb*	83.33%	2.94%	1.67

Structural analysis of the truncated **mstnb** protein revealed marked differences in cysteine residue composition and disulfide bond formation compared with the wild-type protein. The wild-type **mstnb** protein contained 32 cysteine residues, of which three (Cys534, Cys646, and Cys696) were predicted with high confidence to form disulfide bonds, indicating a complex disulfide-bonding network that contributes to structural stability. Several additional cysteine residues were also predicted to participate in disulfide bond formation. In contrast, the truncated **mstnb** protein contained only four cysteine residues. Among these, Cys70 and Cys73 were predicted to have the potential for disulfide bond formation, whereas Cys5 and Cys46 were identified as non-bonded residues. However, no stable disulfide bonds were predicted in the truncated protein. The substantial reduction in cysteine residues and the absence of stable disulfide bonds suggest a loss of structural integrity and reduced protein stability. Collectively, these structural alterations, together with the observed disruptions in secondary and tertiary structure, support the hypothesis that introduction of an upstream premature stop codon in exon 1 abolishes myostatin activity by generating a structurally unstable and non-functional **mstnb** protein.

## Discussion

Myostatin (*mstn*) functions as a key negative regulator of the skeletal muscle development and growth. Myostatin has been shown to be responsible for the double-muscled phenotype, and its role as a negative regulator of muscle development has been validated in multiple fish species through the generation of F_0_ or homozygous mutants [33]. Specifically, *mstnb* suppresses the transcription of *myod1*, a critical gene involved in the myogenic pathway. Disruption of the *mstn* gene in aquaculture species may lead to increased muscle growth, thereby improving traits associated with production [34]. In the present study, donor DNA was designed to include a single nucleotide substitution, that generates an upstream premature stop codon, resulting in the production of a truncated protein. *In silico* analyses were conducted using InterPro and SWISS-MODEL to characterize and compare the domain organization and predicted three-dimensional structures of the wild-type and mutant mstnb proteins.

Genome editing refers to the targeted modification of the genetic material of living organisms including aquaculture species. Advances genome editing field have enabled targeted genomic modifications, including gene disruption through non-homologous end joining (NHEJ) and precise genome editing via homology-directed repair (HDR) in the presence of donor DNA template [35]. Precise genome editing can be achieved through several approaches, including homology-directed repair (HDR), base editing (BE), and prime editing (PE) [36,37]. BE and PE aim to minimize indel formation by employing nickases fused to deaminases or reverse transcriptase, respectively, and are primarily optimized for introducing small, precise genetic modifications [38,39]. However, HDR is better suited for introducing extensive or complex genomic modifications [19]. It functions by inducing DNA double-strand breaks (DSBs) using programmable nucleases, followed by precise repair through homology-directed repair (HDR) using an exogenously supplied donor DNA template [40]. Moreover, exogenously supplied donor DNA templates carrying the desired genetic can be incorporated at into the target locus through HDR, enabling precise genome modification [41,42]. However, in most cellular contexts, DSBs are repaired more frequently through the error-prone non-homologous end joining (NHEJ) pathway than through HDR. To improve HDR efficiency, several strategies have been developed, including cell cycle modulation, optimization of homologous donor template design, and promotion of extensive DNA end resection, a process that is predominantly active during the S and G2 phases of the mitotic cell cycle [43,44]. Among these approaches, donor template construction constitutes the the primary focus of the present study. Several studies have demonstrated the successful application of HDR-mediated knock-in using donor templates for precise genome editing in non-dividing cells [45] including the correction of disease-associated mutations [46], and genome editing of in non-dividing glial cells and neurons to model neurodegenerative diseases in mammalian central nervous system (CNS) models [47].

The present study was designed using an RNA-guided recombinase (RGR)-mediated genome editing, which is mechanistically distinct from conventional homology-directed repair (HDR)-based genome editing systems. HDR-dependent approaches rely on cellular DNA repair machinery to incorporate donor templates following the induction of DNA double-strand breaks (DSBs), whereas the RGR-mediated platform facilitates targeted DNA recombination through programmable recombinase activity [48]. In the present study, the donor construct was designed to facilitate a targeted nucleotide substitution that introduces a premature stop codon in the *mstnb* gene through RNA-guided recombinase (RGR)-mediated genome editing. Desired sequence modifications can be introduced through homology-directed repair (HDR) by delivering an engineered donor DNA template containing homology arms flanking the targeted DNA double-strand break (DSB) generated by nuclease-mediated genome editing [49]. A similar approach has been reported in zebrafish [23], in which a donor DNA template was constructed to facilitate RNA-guided recombinase (RGR)-mediated targeted DNA recombination. Furthurmore, homologous donor DNA templates are typically delivered either as linearized DNA molecules or as circular plasmid. In *Drosophila* embryos, circular plasmid donors (~4.2 kb) exhibited better performance; however, they remain less efficient than linear double-stranded DNA (dsDNA) donors [50]. However, linear dsDNA donors are prone to degradation by exonucleases or conversion into long, linear concatemers, through end-joining activities, rendering them ineffective as donor templates [50,51]. In mammalian cells, plasmid donors containing at least 1–2 kb of total homology are typically employed to introduce large sequence modifications following target site cleavage [51–53]. Accordingly, a 2.3 kb donor DNA template was constructed and validated through Sanger sequencing for use in RGR-mediated targeted genome editing.

Site-directed mutagenesis (SDM) enables precise, targeted modifications including insertions, deletions, and substitutions, in double-stranded DNA facilitating the investigation of genetically encoded changes in protein structure and function. It can also be used to introduce or remove specific DNA sequences that facilitate subsequent genetic manipulation, such as restriction endonuclease recognition sites [54,55]. Engineered mutations can disrupt protein-protein interactions, neutralizing abolish enzymatic activity or alter post-translational modifications [56]. Single point mutations in protein coding sequences can disrupt or alter protein structural conformation potentially leading changes in protein function and consequent phenotypic variation. In addition, site-directed mutagenesis is widely used to introduce specific amino acid substitutions *in vitro*, enabling detailed analysis of protein structure-function relationships [57]. In the present study, site-directed mutagenesis (SDM) was employed to introduce a precise single-nucleotide into the donor DNA template. The mutagenic forward primer was designed to be identical to the parental DNA sequence except for a deliberate mismatch at the target nucleotide, thereby directing the desired base substitution during amplification. Specially,. substitution of the first guanine (G) in the GGA codon with thymine (T) converted the codon to the TGA stop codon successfully demonstrating the generation of a targeted nonsense mutation. Following site-directed mutagenesis, a donor DNA template carrying the intended point mutation was successfully constructed and validated by Sanger sequencing for its application in RGR-mediated genome editing.

While experimental approaches remain the gold standard for assessing the functional effects of protein mutations, they are often costly and time-consuming. Therefore, computational prediction tools are increasingly employed to rapidly evaluate the potential impact of mutations and guide subsequent experimental investigations [58]. *In silico* analyses can predict the structural consequences of protein mutations and help infer their potential clinical significance by elucidating genotype phenotype correlations [59,60]. In the present study, the InterPro database was used to identify conserved domains and functionally important sites in both the wild-type and mutant *Mstnb* proteins. The wild-type *Mstnb* protein retains an intact domain architecture, including the (TGF-β propeptide domain and conserved sites), which are essential for its normal extracellular cytokine/growth factor activity in the negative regulation of muscle growth. In contrast, the mutant Mstnb protein lacks the TGF-β propeptide domain, and retains only the N-terminal signal peptide, with complete lost of the C-terminal domain required for proteolytic processing and cytokine activity. Moreover, the predicted protein structure revealed complete loss of the C-terminal β-sheets-rich region following truncation. This observation was further supported by the comparative pLDDT analysis, which showed a well-defined, high-confidence structured core in the wild-type Mstnb protein, whereas the truncated Mstnb protein exhibited but a disrupted fold with markedly reduced structural confidence.

Structural validation indicated that the wild-type Mstnb proteinpossesses superior stereochemical quality compared with the truncated model. As shown by the Ramachandran plot (Fig 7), 89.28% of residues in the wild-type protein were located in the most favoured regions, compared with only 83.33% in the truncated protein. Consistent with these findings, the wild-type *Mstnb* protein exhibited a lower MolProbity score (1.38) than the truncated protein (1.67), indicating fewer geometric errors and higher overall structural quality in the wild-type model [61]. Moreover, the QMEAN Z-score indicated structural destabilization of the truncated *Mstnb* protein model as its score deviated further from those of experimentally resolved protein structures in the Protein Data Bank (PDB), suggesting reduced structural reliability [62,63]. The structural stability of a protein is largely dependent on the formation of disulphide bonds between cysteine residues [64]. Analysis of disulphide bond formation revealed that the wild-type *Mstnb* protein contains multiple cysteine capable of forming disulphide bonds and maintaining a stable three-dimentional (3D) conformation. In contrast, the truncated *Mstnb*-protein exhibited a marked reduction in cysteine residues, resulting in reduced predicted disulphide bond formation and likely disrupting the formation of the conserved cysteine-knot motif. This may like to result in improper protein folding and reduced structural stability of the truncated *Mstnb* protein. Consequently, the impaired folding and reduced structural stability of the truncated *Mstnb* protein are likely to compromise the receptor interaction, disrupt downstream signaling pathways, and ultimately diminish its biological activity.

## Conclusion

In the present study, an engineered donor DNA template was constructed using site-directed mutagenesis (SDM) to introduce a targeted mutation into exon 1 of the *mstnb* gene in *L. rohita* for RNA-guided recombinase (RGR)-mediated genome editing. A targeted G > T substitution was designed to generate a premature upstream stop codon, and successful incorporation of the mutation was confirmed by Sanger sequencing. The introduced mutation was predicted to produce a truncated Mstnb protein. Comprehensive *in silico* analyses revealed that the truncation resulted in the loss of functional domains, structural destabilization, reduced stereochemical quality, diminished disulfide bond formation, and disruption of the conserved cystine-knot motif. Furthermore, comparative structural analyses, including pLDDT, Ramachandran plot, MolProbity, and QMEAN Z-score assessments consistently demonstrated compromised structural integrity and reduced model quality of the truncated Mstnb protein relative to the wild-type protein. These structural alterations are predicted to impair protein folding, receptor interactions, downstream signaling, and ultimately the biological activity of Mstnb. Collectively, thesefindings provide a strong foundation for subsequent *in vitro* and *in vivo* validation of the engineered donor construct and demonstrate its potential for precise RGR-mediated genome editing in *L. rohita* and other aquaculture species. The validated donor DNA construct offers a valuable resource for RGR-mediated targeted knockout of the *mstnb* gene in *L. rohita* and provides a robust framework for future precision genome-editing applications at the *mstnb* locus.

## Supporting information

S1 Fig Sanger sequencing chromatogram showing successful integration of the target sequence, as confirmed by the presence of the expected nucleotide sequence at the target site. (TIFF)

S2 Fig Chromatograms obtained from Sanger sequencing following site-directed mutagenesis (SDM), showing successful introduction of the desired mutation. The sequencing traces display a clear thymine (T) peak at the mutation site, confirming the replacement of the original guanine (G) nucleotide.
(TIFF)
