# Supplementary figures and images for: Design and in silico validation of donor DNA for RNA-guided recombinase-mediated knockout of mstnb gene in Labeo rohita
Source: PLoS One. 2026 Jul 15;21(7):e0352166. doi: 10.1371/journal.pone.0352166 (PMC13372240; doi:10.1371/journal.pone.0352166)

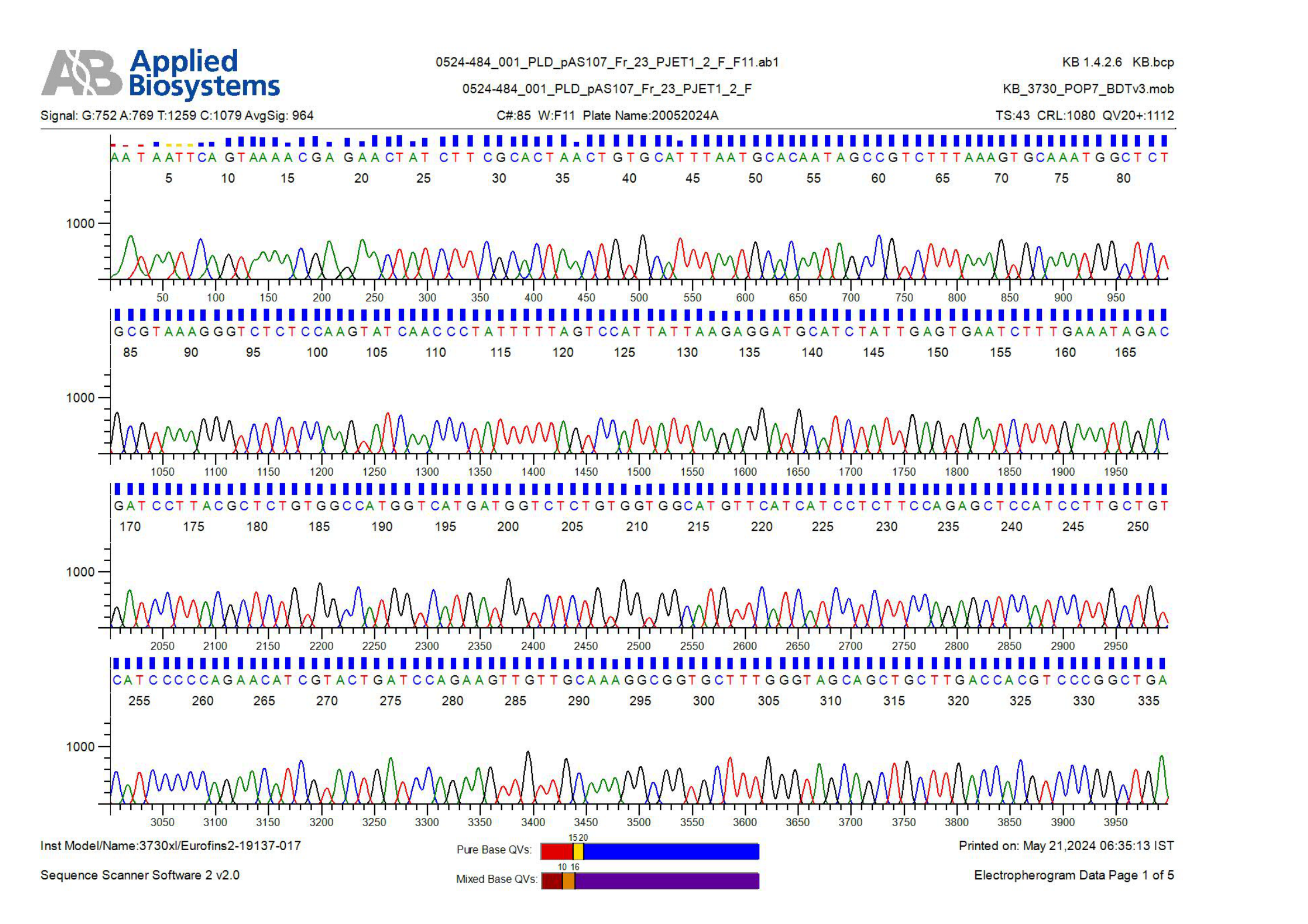

Supplement: S1 Fig — (TIFF) [file pone.0352166.s001.tiff]

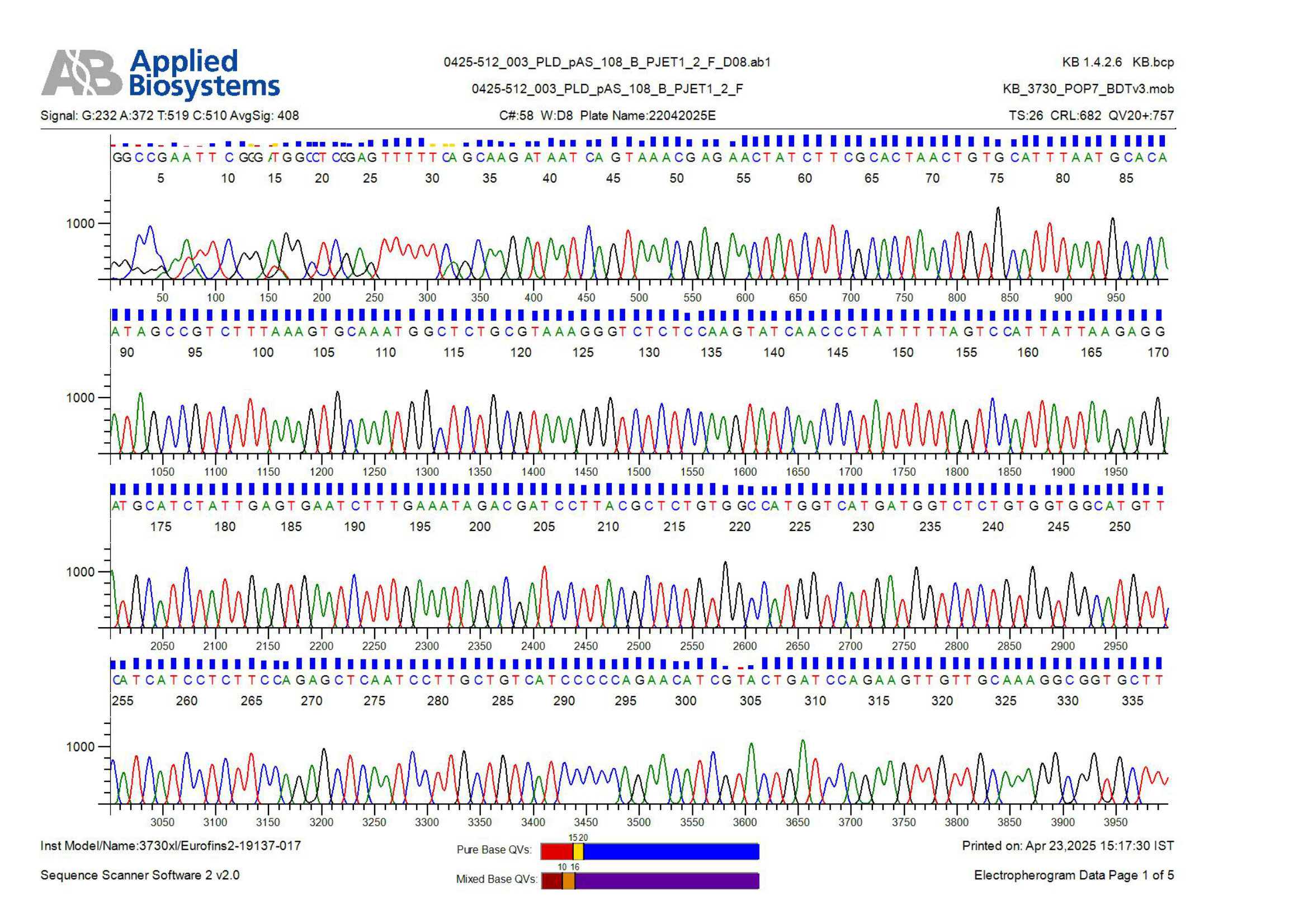

Supplement: S2 Fig — The sequencing traces display a clear thymine (T) peak at the mutation site, confirming the replacement of the original guanine (G) nucleotide. (TIFF) [file pone.0352166.s002.tiff]
